# RNA-Seq analysis and comparison of corneal epithelium in keratoconus and myopia patients

**DOI:** 10.1038/s41598-017-18480-x

**Published:** 2018-01-10

**Authors:** Jingjing You, Susan M. Corley, Li Wen, Chris Hodge, Roland Höllhumer, Michele C. Madigan, Marc R. Wilkins, Gerard Sutton

**Affiliations:** 1 Save Sight Institute, Sydney Medical School, University of Sydney, Sydney, Australia; 2 School of Optometry and Vision Science, University of New South Wales, New South Wales, Australia; 3 School of Biotechnology and Biomolecular Science, NSW System Biology Initiative, University of New South Wales, New South Wales, Australia; 4 Lions NSW Eye Bank, Sydney, Australia; 5 Vision Eye Institute, Chatswood New South Wales, Australia; 6 University of the Witwatersrand, Johannesburg, South Africa; 7 The Cornea Foundation, Johannesburg, South Africa

**Keywords:** RNA sequencing, Corneal diseases

## Abstract

Keratoconus is a common degenerative corneal disease that can lead to significant visual morbidity, and both genetic and environmental factors have been implicated in its pathogenesis. We compared the transcriptome of keratoconus and control epithelium using RNA-Seq. Epithelial tissues were obtained prior to surgery from keratoconus and myopia control patients, undergoing collagen cross-linking and photorefractive keratectomy, respectively. We identified major differences in keratoconus linked to cell-cell communication, cell signalling and cellular metabolism. The genes associated with the Hedgehog, Wnt and Notch1 signaling pathways were down-regulated in keratoconus. We also identified plasmolipin and Notch1 as being significantly reduced in keratoconus for both gene and protein expression (p < 0.05). Plasmolipin is a novel protein identified in human corneal epithelium, and has been demonstrated to have a key role in epithelial cell differentiation in other tissues. This study shows altered gene and protein expression of these three proteins in keratoconus, and further studies are clearly warranted to confirm the functional role of these proteins in the pathogenesis of keratoconus.

## Introduction

Keratoconus (KC) is a progressive corneal degenerative disease with a yet to be fully elucidated etiology and pathogenesis. KC progression leads to an irregular-shaped cornea that can significantly affect visual function and significantly impact on the patient’s quality of life^1^. The incidence of KC has been reported to be as high as 2.34% in a general population^2^. With onset typically at puberty, KC represents a life-long consideration for affected patients. Early diagnosis and appropriate treatment is essential for optimal rehabilitation. Histological examination shows that changes are predominantly seen in epithelium, Bowman’s layer and stroma in KC corneas^3^. Whether the initial changes occur first in the epithelium or stroma remains unclear. Early histopathological studies highlighted the initial abnormalities in the corneal epithelium and it was postulated that insults to the epithelium led to a release of proteolytic enzymes, that degraded the stromal tissue with an ensuing cascade of tissue damage^4,5^. We previously identified novel abnormalities in the Wnt signaling pathway in the epithelium of KC patients^6–8^. While many researchers have proposed abnormal stromal metabolism as the primary site of metabolic dysfunction, there is no doubt that the presence of a healthy corneal epithelium is essential to stromal keratocyte function and survival^9–12^. The corneal epithelium is the outermost layer of the cornea which acts as a physical barrier to pathogens and is in contact with the tear film. The epithelium is stratified into three cellular layers; basal cells, wing cells and superficial cells^13^. The corneal epithelium is constantly being renewed as new epithelial cells are generated at the basal level from the limbus (the border of the cornea), and then transformed into wing cells as they migrate anteriorly towards the surface of the cornea to form the superficial squamous cells that eventually lose their adhesion attachments (desmosomes) and are sloughed off into the tear film^13,14^. In this way, the entire corneal epithelium is replaced approximately every 7 days^13–15^. Loss of epithelium causes keratocyte apoptosis, and an abnormal epithelium could therefore lead to impaired keratocyte function and collagen synthesis^16–18^. This highly dynamic tissue is affected in KC showing abnormal morphology including epithelial thinning, elongated and irregular shaped basal epithelial cells and breaks in the basement membrane^4^.

Both environmental and genetic factors are thought to be involved in the development of the condition^19,20^. Multiple studies have demonstrated environmental risks for KC, with eye rubbing and atopy considered the most significant factors following multivariate analyses^19–21^. The genetic contribution to KC is more complex. Linkage studies have isolated numerous candidate genetic loci but few have been confirmed by independent studies, highlighting the significant genotypic variation within the disease^22^. Associations with multi-system syndromes further suggest a genetic contribution^23,24^.

Genome-wide association studies (GWAS) for KC have generated mixed results. The first GWAS investigation, which compared 222 affected individuals to several thousand controls, found that no genomic variations reached genome-wide significance level (p < 5 × 10^−8^)^25^. However, certain single nucleotide polymorphisms (SNPs), including *RAB3GAP1* (Rab3 GTPase-activating protein catalytic subunit) SNPs *rs4954218* and *rs6730157* (p = 1.4 × 10^−6^ and 3.4 × 10^−6^ respectively) were confirmed in validation cohorts, suggested *RAB3GAP1* as a potential causative gene for KC^25^. Later GWAS studies identified significant associations between various SNPs of *FOXO1* ^26^, *FNDC3B* ^26^ and *HGF* ^27–29^ with KC.

GWAS findings provide valuable insights as to the possible pathogenesis of KC, however their capacity to provide functional information about the disease is limited. Understanding gene and protein expression patterns in KC is essential for discovering the specific pathways that may be affected in the disease. In the current study we examined, for the first time, the gene expression profiles of corneal epithelium in KC and myopia samples, using an RNA-Seq analysis approach..

## Results

### Gene expression in control corneal epithelial samples

We analysed the transcriptome in 10 control epithelial samples and found that 46249 genomic features (protein coding genes and non-coding RNA genes such as IncRNA, pseudogenes and antisense) had at least one assigned read in the 10 samples tested. Features with low expression were then filtered out, to capture only those genes which were expressed in each of the 10 control samples with at least a minimum of 8 reads per gene. This filtering reduced the number of features to 13308. Of these, 11655 were protein-coding genes and 1643 were non-coding features including lincRNA, antisense transcripts and pseudogenes. Details of these features can be found in Table S1.

The genes with the highest expression (top 2% ranked by RPKM) included: Keratin 3 (KRT3), Keratin 5 (KRT5), Keratin 12 (KRT12), aldehyde dehydrogenase 3 family member A1 (ALDH3A1), clusterin (CLU) and enolase 1 (ENO1). KRT3 and KRT12 are markers for differentiated corneal epithelial cells and are only expressed in the central corneal regions^9^.

### A sex effect is seen for gene expression in the corneal epithelium

**Figure 1:**
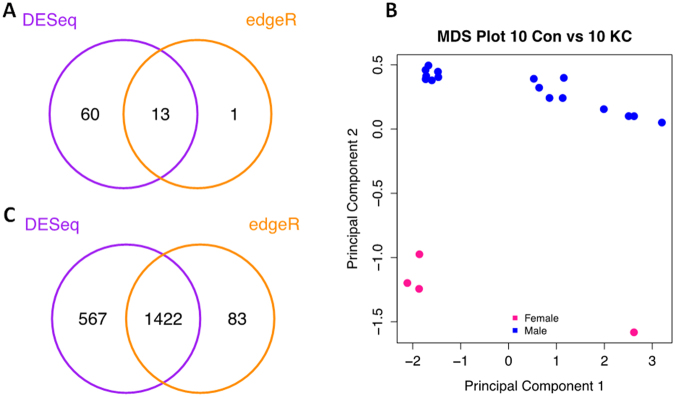
Mulitdimensional Scaling (MDS) analysis of RNA-Seq data demonstrated a sex effect for the samples and number of significantly differentially expressed genes (DEGs) detected. (**A**) Venn diagram of differentially expressed genes found by edgeR and DESeq2 (FDR 0.1) using all 20 samples (10 Control and 10 KC). (**B**) MDS plot of 20 samples shows distinct clustering of samples by sex, female (pink) and male (blue). (**C**) Venn diagram of differentially expressed genes found by edgeR and DESeq2 (FDR 0.1) using only male samples (6 Control and 10 KC).

We initially performed differential gene expression testing on all 10 controls versus 10 KC samples (sample information can be found in Table 1). This identified 14 (edgeR) or 73 (DESeq. 2) differentially expressed genes (DEGs) with a HGNC gene symbol and at FDR = 0.1 (Fig. 1A). These numbers reduced to 4 and 15 for edgeR and DESeq. 2 respectively at FDR = 0.05. However, we noted that the 10 controls included 6 males and 4 females whereas the 10 KC samples were all male. A multidimensional scaling (MDS) plot revealed a clear sex effect with all four female samples clustering away from the male samples (Fig. 1B).

**Table 1:** Corneal epithelial samples used in this study including conditions, sex, age and analysis performed.

Sample ID	Condition	Sex	Age	Analysis	RNA yield (μg)
1	KC	M	17	RNA-Seq	2.80
2	KC	M	39	RNA-Seq	8.48
3	KC	M	40	RNA-Seq	4.32
4	KC	M	22	RNA-Seq	9.13
5	KC	M	28	RNA-Seq	5.24
6	KC	M	16	RNA-Seq	8.65
7	KC	M	25	RNA-Seq	10.02
8	KC	M	22	RNA-Seq & qPCR	5.16
9	KC	M	26	RNA-Seq & qPCR	6.29
10	KC	M	35	RNA-Seq	2.56
11	Control	F	35	RNA-Seq & qPCR	7.21
12	Control	M	32	RNA-Seq & qPCR	7.88
13	Control	F	39	RNA-Seq & qPCR	9.33
14	Control	F	35	RNA-Seq	8.60
15	Control	M	30	RNA-Seq	7.82
16	Control	M	33	RNA-Seq	4.60
17	Control	M	27	RNA-Seq & qPCR	13.59
18	Control	M	43	RNA-Seq	4.83
19	Control	F	23	RNA-Seq	4.69
20	Control	M	23	RNA-Seq & qPCR	4.98
21	Control	F	44	qPCR	6.13
22	Control	F	34	qPCR	7.70
23	Control	F	46	qPCR	6.73
24	Control	F	36	qPCR	5.92
25	Control	F	35	qPCR	4.69
26	KC	F	43	qPCR	2.76
27	KC	M	13	qPCR	5.24
28	KC	M	21	qPCR	3.29
29	KC	M	17	qPCR	5.02
30	KC	M	32	qPCR	9.45
31	KC	M	18	qPCR	8.61
32	KC	M	20	qPCR	7.63
Western blot analysis					
33	KC	F	26	Notch1, Src	
34	KC	M	24	Notch1, Src	
35	KC	M	30	Notch1, NICD, PLLP, Src	
36	KC	F	38	Notch1, NICD, PLLP, Src	
37	KC	M	23	Notch1, NICD, PLLP, Src	
38	KC	F	36	Notch1, NICD, PLLP	
39	KC	M	24	Notch1, NICD, PLLP	
40	KC	M	19	NICD, PLLP	
41	KC	F	24	NICD, PLLP	
42	KC	M	22	NICD, PLLP	
43	KC	M	28	NICD, PLLP	
44	KC	F	25	NICD, PLLP	
45	KC	M	18	NICD, PLLP	
46	control	F	31	NICD, PLLP	
47	control	F	37	NICD, PLLP	
48	control	F	39	NICD, PLLP	
49	control	M	47	Notch1, NICD, PLLP, Src	
50	control	F	45	Notch1, Src	
51	control	F	29	Notch1, Src	
52	control	F	29	NICD, PLLP, Src	
53	control	M	32	Notch1, NICD, PLLP, Src

### Differential expression in Keratoconus excluding sex effect

Given the clear sex effect and the fact that all our KC samples were male we re-analysed the data using only the male controls (n = 6) and male KC samples (n = 10). Using edgeR we obtained 1505 DEGs and DESeq2 found 1989 DEGs at FDR = 0.1 (Fig. 1C). These numbers reduced to 442 and 670 for edgeR and DESeq. 2 respectively at FDR = 0.05. There was substantial overlap in the two sets of DEGs with 94% of the edgeR set being found by DESeq. 2 at an FDR = 0.1 as shown in the Venn diagram in Fig. 1C. We found that 79% of DEGs were down-regulated in KC in both the edgeR and DESeq. 2 sets with a cut-off of FDR = 0.1. Details of the DEGs can be found in Table S2. We used the results of the differential expression analysis excluding the female samples in all downstream analysis. We have conducted our downstream analysis on the edgeR results using the downstream analysis functions which are associated with this method. This allows a seamless workflow and the use of tools such as the gene set analysis functions which require the input of the entire list of genes tested for differential expression.

### Gene Ontology Enrichment

To understand the biological function of the DEGs, we applied the goana function in the limma Bioconductor package to the differentially expressed genes identified by edgeR to find statistically significant enriched gene ontology terms, using an FDR cut-off of 0.05. The top 10 terms in each of the Gene Ontology categories [Biological process (BP), Molecular function (MF) and Cellular component (CC)] are set out in Table 2. This analysis indicated that the differentially expressed genes were strongly associated with the cell surface, plasma membrane and cell junctions (Table 2, CC). Genes associated with synapses were enriched and showed upregulation in KC (Table 2, CC Upreg). The most significant down-regulated BP terms in KC were cell to cell communication followed by two signaling related GO terms. The upregulated BP terms in KC included ion transport and eye development. For MF terms, genes associated with GTPase regulation were downregulated and membrane transporter activity were upregulated consistent with ion transport in the BP category. The top 100 gene ontology terms in each of the BP, MF and CC classifications can be found in Table S3.

**Table 2:** Top ten GO terms identified from DEGs associated with KC.

**BP Downreg**	**BP Upreg**
cell communication	ion transport
single organism signaling	nephric duct morphogenesis
signaling	resolution of recombination intermediates
positive regulation of cell projection organization	camera-type eye development
signal transduction	eye development
regulation of cell projection organization	nephric duct development
animal organ development	metal ion transport
cellular response to stimulus	multicellular organismal signaling
cell surface receptor signaling pathway	neuron projection development
regulation of hydrolase activity	respiratory gaseous exchange
**MF Downreg**	**MF Upreg**
molecular function regulator	metal ion transmembrane transporter activity
GTPase regulator activity	calcium-transporting ATPase activity
GTPase activator activity	active transmembrane transporter activity
enzyme regulator activity	ion transmembrane transporter activity
molecular transducer activity	inorganic cation transmembrane transporter action
receptor activity	filamin binding
nucleoside-triphosphatase regulator activity	substrate-specific transmembrane transporter
Rho guanyl-nucleotide exchange factor activity	molecular transducer activity
protein self-association	receptor activity
kinase binding	cation transmembrane transporter activity
**CC Downreg**	glycerol-3-phosphate dehydrogenase activity
cell periphery	**CC Upreg**
plasma membrane	plasma membrane part
membrane	synaptic membrane
intrinsic component of membrane	integral component of plasma membrane
adherens junction	intrinsic component of plasma membrane
membrane part	plasma membrane
anchoring junction	cell periphery
integral component of membrane	postsynaptic membrane
vesicle	synapse part
plasma membrane part	plasma membrane region

### Gene set analysis to find enriched KEGG pathways

The gene set analysis methodology considers the entire list of genes tested for differential expression rather than using the DEGs derived from an arbitrary FDR cut-off or fold change threshold. We used the fry function (limma Bioconductor package) to perform gene set analysis on the genes tested using edgeR (n = 14585). We used an FDR cut-off of 0.01 for this analysis and found 86 KEGG gene sets that tend to be differentially expressed without regard for direction of the change (up-regulation or down-regulation). Details of the 86 KEGG pathways can be found in Table S4. The most statistically significant pathways include major pathways involved in cell differentiation (Hedgehog signaling, N = 29)^30^, cell migration (Wnt signaling, N = 114)^31^, and cell-to-cell communication (Notch signaling, N = 39)^32^.

### Comparing the effect of Keratoconus on corneal epithelium versus the entire corneal button

Kabza *et al*., 2017 compared the transcriptome in the cornea of patients with KC with patients undergoing corneal transplant for other reasons such as bullous keratopathy, corneal scarring, corneal ulcers and perforations. As this study also used RNA-Seq and attempted to explain the features of KC using this approach, it is important to compare it to our studies. We took the count tables available at GSE77938 (GSE77938 discovery gene counts.txt and GSE77938 replication gene counts.txt) and used edgeR to carry out differential expression on this data set, using the same parameters in our study, as described in Methods.

Firstly, we found that the controls in the Kabza *et al*., 2017 data had greater variation than the KC samples, with a higher biological coefficient of variation (BCV) as calculated using edgeR (Kabza *et al*., Controls: BCV = 0.57, KC: BCV = 0.41). In contrast, our controls were more similar to each other (Controls: BCV = 0.41, KC: BCV = 0.56). Around half of our DEGs were also identified in the Kabza *et al*. data (217/442, edgeR, FDR = 0.05). Of the 217 common DEGs identified by edgeR, 92 changed in the same direction while 125 changed in the opposite direction when compared between studies.

**Figure 2:**
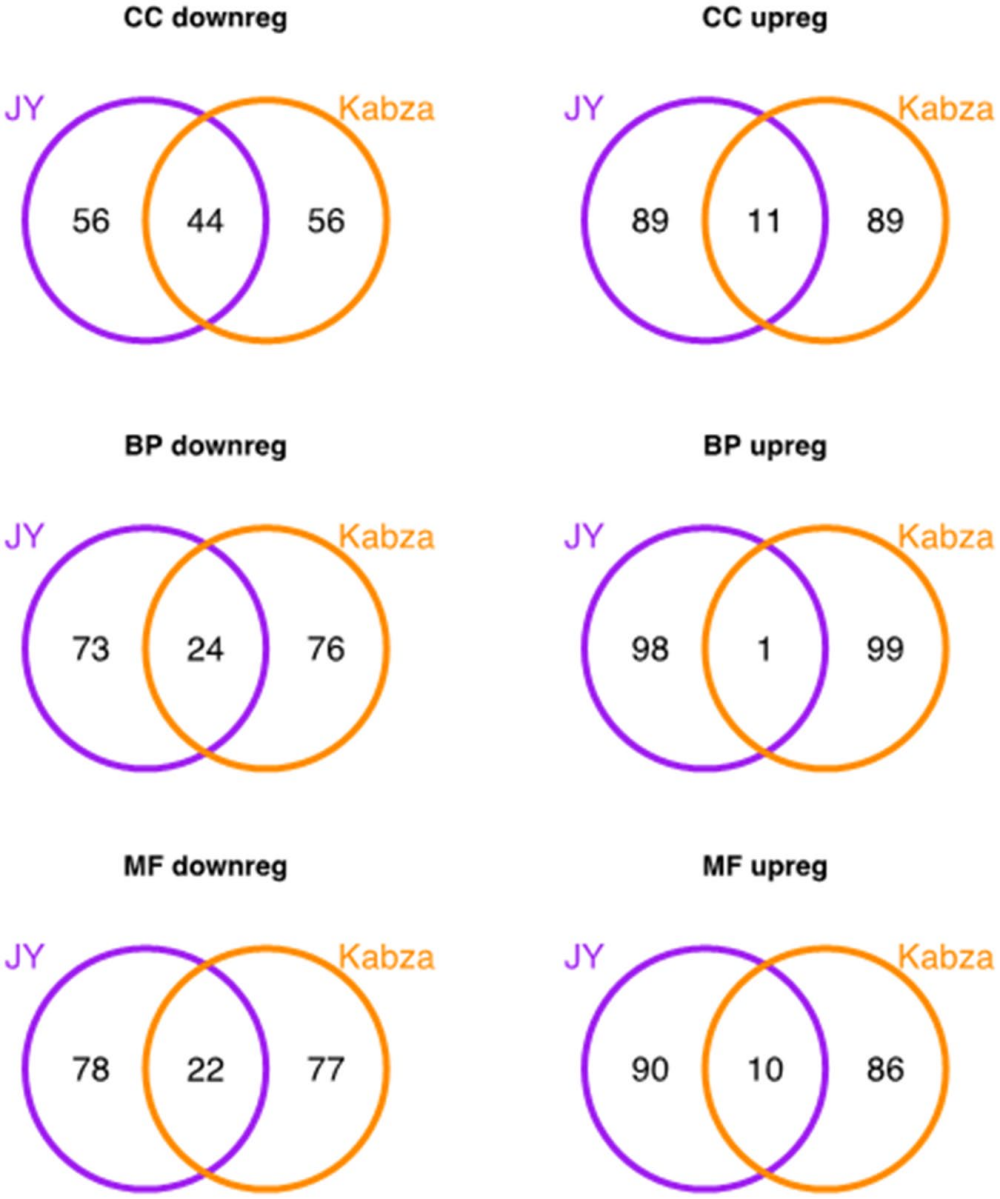
Venn diagrams of the most enriched gene ontology terms in the edgeR DEGs derived in our study (JY) and in the edgeR DEGs generated from our analysis of the Kabza *et al.* data (Kabza *et al.* 2017). Enriched gene ontology terms in the three categories, CC: cellular component, BP: biological process, and MF: molecular function, were found using the goana function (limma).

Comparisons of functional categories in our DEGs and those arising from the Kabza *et al*. data showed that most overlap was seen in the CC classified GO terms which were down-regulated in the KC (44% overlap, Fig. 2), these terms included “cell periphery, plasma membrane, adherens junction, anchoring junction, vesicle, focal adhesion”. The least overlap was seen in the BP classified GO terms in the KC up-regulated genes, where there was only one common term, “transmembrane transport”. The detailed comparative analysis can be found in Table S5.

We used the same gene set analysis methodology to highlight significant KEGG pathways in the Kabza *et al*. data set. We found 177 KEGG gene sets had genes which tended to be differentially expressed (FDR < 0.01). Of these, 18 KEGG pathways contained genes which tend to be up-regulated, 107 contained genes which tended to be down-regulated and the remaining 52 KEGG pathways contained genes which tended to be differentially expressed without regard to direction. Comparison with our study of the corneal epithelium reveals an overlap of 85 KEGG pathways included several signaling pathways (Hedgehog, Wnt, Notch, MAPK and GNRH) as well as cytokine-cytokine receptor interaction, regulation of the actin cytoskeleton and tight junction, focal adhesion and gap junction, mismatch repair and valine, leucine and isoleucine degradation. The enriched KEGG pathways from our analysis and our additional analysis of the Kabza *et al*. data can be found in Table S4.

Some caution needs to be exercised in the interpretation of this comparison of our results with the data generated by Kabza *et al*. given the different nature of the control samples used in both studies. The controls used in the Kabza *et al*. study were undergoing corneal transplant as a result of conditions such as bullous keratopathy, corneal scarring, corneal ulcers and perforations, whereas our controls were only affected by myopia (a refractive condition). It is plausible that the corneal health of the controls used in the Kabza *et al*. study was more severely compromised than that of the KC samples. In particular, it is possible that the differential results from the Kabza *et al*. study were related to non-KC disease.

### qPCR and Western blot analysis

**Figure 3:**
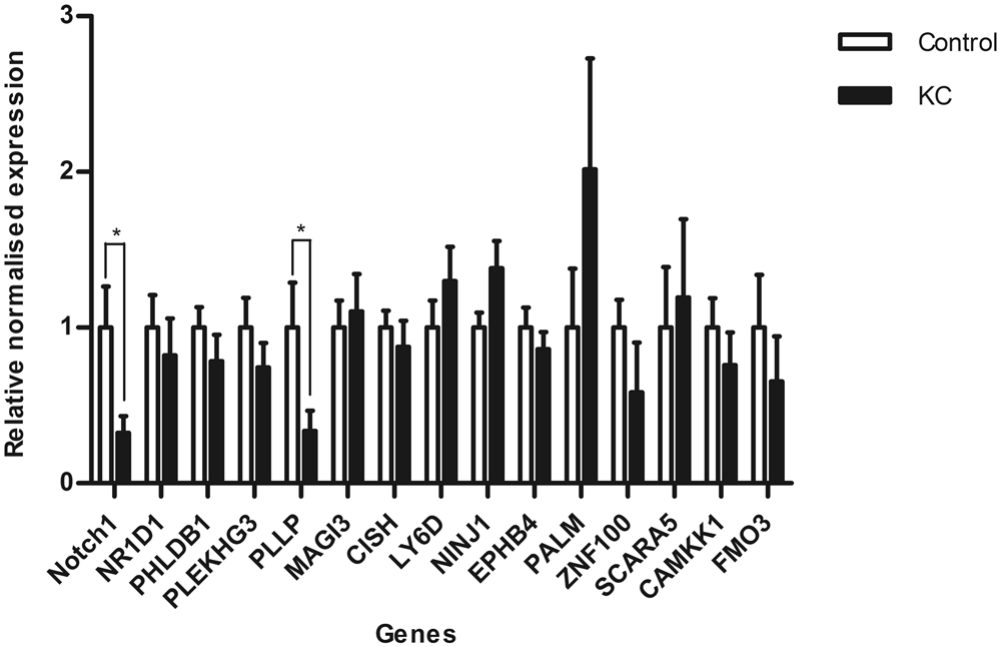
Fifteen genes were validated by qPCR with *Notch1* and *PLLP* being significantly down-regulated in KC compared to controls (*p < 0.05).

**Figure 4:**
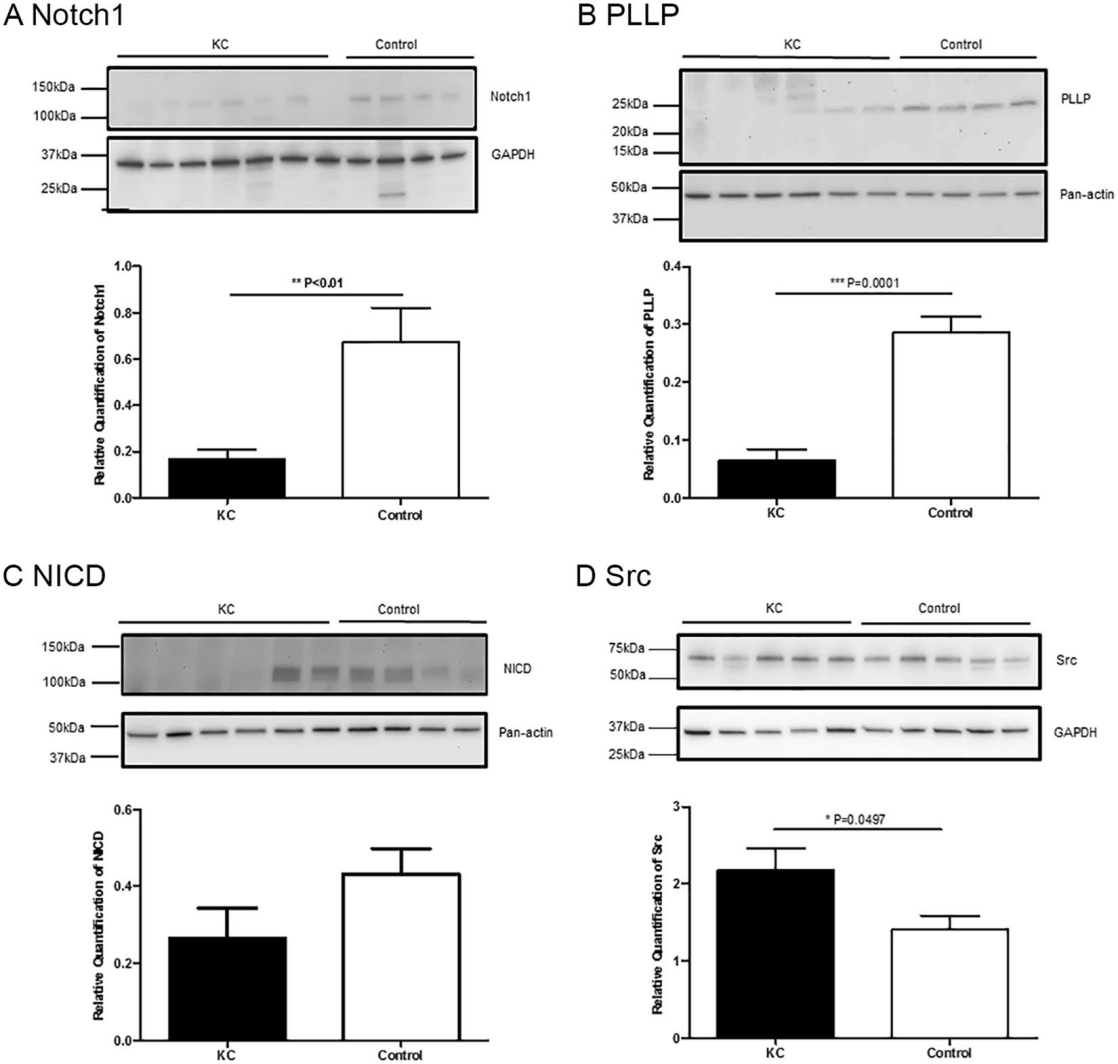
Cropped reprentative of Western blot images showing the detection of bands at the reported molecular weight (kDa) for each protein. Relative quantification of immunoblots using GAPDH and Pan-actin as reference proteins showed a significant reduction of Notch1 and PLLP protein expression in KC compared to controls (**A** and **B**, p < 0.01). No significant difference was found between KC and controls for relative protein expression levels of NICD using Pan-actin as a reference protein (**C**). Relative quantification of Src expression using GAPDH as a reference protein, showed a significant increase in KC samples compared to control samples (**D**) (p = 0.0497). Full-length blots of each tested protein are in Supplementary Figure S1.

We compared our RNA-Seq results with RNA expression using real time qPCR. For this we selected fifteen DEGs involved in a range of functions including cell-cell communication, transport and metabolism (Table 3). Of the genes tested by qPCR, *PLLP* (average Cq = 28.5) and *Notch1* (average Cq = 31.4) were significantly reduced in KC compared to controls (fold change > 3, p < 0.05, Fig. 3). Four of the 15 genes, *zinc finger protein 100* (*ZNF100*), *scavenger receptor class A member 5* (*SCARA5*), *flavin-containing monooxygenase 3* (*FMO3*) and *calcium/calmodulin dependent protein kinase kinase 1* (*CAMKK1)*, had average Cq value above 33 cycles, indicating low expression and increasing the difficulty of detecting a difference in expression between conditions. Protein expression of PLLP and Notch 1 were also found significantly reduced in KC and control samples, consistent with qPCR results (Fig. 4). In contrast, Src (proto-oncogene, non-receptor tyrosine kinase; also called c-Src) showed a significant up-regulation in KC compared to control group (p = 0.0497), and no significant difference of NICD (intracellular domain of the notch protein) expression between KC and control groups was detected (Fig. 4).

**Table 3:** Fifteen genes selected for qPCR analysis.

**Gene Symbol**	**Ensembl Gene ID**	**Relevant GO terms**
NOTCH1	ENSG00000148400	Negative regulation of transcription from RNA polymerase II promoter;Angiogenesis; In utero embryonic development; Cell fate specification; Epithelium to mesenchymal transition
PLLP	ENSG00000102934	Membrane raft polarization; Transport; Ion transport; Protein localization; Response to wounding
LY6D	ENSG00000167656	Metabolism of proteins; B Cell Development Pathways; Post-translational modification
CAMKK1	ENSG00000004660	Protein phosphorylation; Intracellular signal transduction
CISH	ENSG00000114737	Regulation of cell growth; Negative regulation of protein kinase activity; Protein kinases C-activating G-protein coupled receptor signaling pathway; Negative regulation of signal transduction; Protein ubiquitination
PLEKHG3	ENSG00000126822	Regulation of Rho protein signal transduction; Positive regulation of GTPase activity
NR1D1	ENSG00000126368	Negative regulation of transcription from RNA polymerase II promoter; Glycogen biosynthetic process; Transcription, DNA-templated; Transcription initiation from RNA polymerase II promoter
NINJ1	ENST00000375446	Positive regulation of cell-matrix adhesion; Cell adhesion; Nervous system development; Tissue regeneration; Hyaloid vascular plexus regression
Scara5	ENSG00000168079	Transport; Ion transport; Cellular iron ion homeostasis; Endocytosis; Receptor-mediated endocytosis
EPHB4	ENSG00000196411	Angiogenesis; Cell migration involved in sprouting angiogenesis; Heart morphogenesis; Protein phosphorylation; Cell adhesion
MAGI3	ENSG00000081026	Apoptotic process; Signal transduction; Viral process; Phosphorylation; Intracellular signal transduction
PHLDB1	ENSG00000019144	Regulation of gastrulation; Regulation of epithelial to mesenchymal transition; Regulation of microtubule cytoskeleton organization; Positive regulation of basement membrane assembly involved in embryonic body morphogenesis
PALM	ENSG00000099864	Movement of cell or subcellular component; Cytoskeletion organization; Negative regulation of adenylate cyclase activity; Protein localization; Regulation of cell shape
FMO3	ENSG00000007933	Xenobiotic metabolic process; Oxidation-reduction process
ZNF100	ENSG00000197020	Transcription, DNA-templated; Regulation of transcription, DNA-templated

## Discussion

In this study, we examined whether gene expression in corneal epithelium is affected by KC. RNA-Seq was used to compare the global gene expression in the surgically removed corneal epithelial tissues from KC patients with epithelium of myopia patients (control group). A database of both mRNA and non-coding RNA expressed in corneal epithelial tissue was also generated, which is a first in this field.

### Gene profiling of human corneal epithelium

RNA-Seq is a powerful technique for identifying gene expression over a broad dynamic range. To date there is only one published high throughput screening of corneal epithelial gene expression^33^. This microarray study found evidence for the expression of 8905 transcripts in the corneal epithelium (48% of the total genes in microarray)^33^. By comparison, using our 10 control samples we detected 46,249 genomic features of which 13308 were expressed in all 10 samples with a minimum of 8 reads per gene. Our study has generated the most complete expression profile to date of corneal epithelium.

### Sex effect and functional analysis of DEGs in KC

Our initial differential expression analysis indicated a sex effect on gene expression in the human corneal epithelium. This is consistent with earlier work showing sex-related differences in gene expression of human corneal epithelial cells^34^. Furthermore, it matches clinical registry findings which suggest a predisposition of males to develop KC^2,35,36^. Since our KC cohort were all male, to achieve a balanced design it was necessary to exclude the 4 female samples from the control set and to proceed with this analysis using only the male samples.

Our DEG analysis showed that around 80% of DEGs were decreased in KC compared to controls. Functional analysis of these DEGs suggested the top cellular components (CC terms) are relevant to the cell periphery, the plasma membrane and junctions between cells. In terms of biological process, the DEGs are most strongly associated with cell communication, signaling, eye development, migration, cell-cell adhesion, ion transport, cell apoptosis and regulation of hydrolase activity. In particular, our study suggests that the barrier function of the corneal epithelium may be compromised in KC, given that DEGs in categories of tight junctions, gap junctions, adherens junction, focal adhesion, cell-cell communication and signaling were down-regulated. These functions are important in the healthy corneal epithelium which is constantly undergoing renewal through the processes of cell division, migration and differentiation. These functional classifications are consistent with histological observations of KC epithelium, which typically appear flattened and thinned, with fewer layers and diminished architecture of the basal and wing cell layers, associated with changes in cell-cell junctions and loss of cell polarity^3,5,37^. The regulation of hydrolase activity category was also down-regulated, however the hydrolase-related DEGs identified in our study did not include the previously suggested MMPs and TIMPs^20^.

Interestingly, we see a similarity in the functional categories of the DEGs identified in the current study and the functions of genes highlighted by earlier GWAS studies as likely being involved in KC. It has previously been suggested that *RAB3GAP1* is potentially causative in KC^25,38^. *RAB3GAP1* is a direct activator of Rab3 GTPase, a key regulator of calcium-mediated hormone and neurotransmitter exocytosis. Although *RAB3GAP1* was not in our DEG list, we found enrichment in GTPase regulation in our differentially expressed genes with 21 of the edgeR DEGs having this gene ontology annotation. All of these genes are subsequently included in the set of 57 DEGs involved in hydrolase regulation. The GTPases play an important role in signal transduction, cell differentiation, transport of vesicles in cells and translocation of proteins through membranes^39^. The Rho family of GTPases has been associated with corneal epithelial migration, focal adhesion formation and cell junction assembly^40,41^. Corneal wound healing mechanisms have been found to be driven by GTPases, which regulate the actin cytoskeleton, cell migration and proliferation^42^, and similarities of molecular alterations between the corneal wound healing cascade and KC have been previously reported^43^.

### Potential pathways associated with KC

The GO analysis and gene set analysis of KEGG pathways together point to dysregulation of signalling (top down-regulated Biological Process GO terms) in particular Hedgehog signalling, Wnt signalling and Notch1 signalling (top down-regulated KEGG gene sets). We have previously suggested a role for Wnt signalling in KC^6,7^. In addition, our GO and pathway analysis shows down-regulation of genes associated with cell communication, the cell cycle, migration and differentiation and the metabolism of arachidonic acid, chondroitin sulphate and unsaturated fatty acids. We also see dysregulation of autophagy which is involved with degradation of unnecessary or dysfunctional molecules in cells which may be relevant to abnormal apoptotic mechanisms^44^. Apoptosis, inflammation and oxidative stress have been proposed to be associated with KC development, and analysis of the KC epithelial and stromal proteome found altered protein expression associated with tissue structure, metabolic and oxidative stress; and down regulation of proteins involved in proliferation, growth and migration^45^. We found GO terms associated with up-regulated genes included functions involving synapses, suggesting possible alterations in epithelial corneal nerves. *In vivo* laser scanning confocal microscopy also showed that the nerve morphology in KC is significantly different to normal cornea, with reduced central sub-basal nerve density, thicker nerve fibres and altered nerve fibre orientation^46^. The upregulated pathways in KC suggest the initiation of repair mechanisms both of DNA damage (DNA mismatch repair pathway) and of cellular damage (upregulation of cell cycle, Jak Stat signalling, TGF beta signalling and Nod-like receptor signalling pathways). Taken together these findings highlight the complexity of underlying and compensatory mechanisms seen in KC.

### Comparison to a RNA-Seq study of KC using the entire corneal button

We directly compared our results to a recently published RNA-Seq study of KC corneas^47^. This study differed from ours in two major respects: 1. full thickness corneal buttons removed from KC patients were used, with RNA extracted from all corneal layers, and 2. the control group included corneas from various non-KC pathologies including bullous keratopathy, corneal scarring, ulcers and perforations. Our controls consisted of mildly myopic participants, with no evidence of corneal tissue injury, damage or chronic pathology. Although many KC patients are also myopic we believe our control cohort represents a more robust and appropriate control set due to the absence of corneal pathology. We found far fewer DEGs in our study comparing the corneal epithelium of KC with myopic patients; approximately half of the DEGs found in our study were also found using the Kabza *et al*. data. However the direction of gene expression change was not always consistent between the two studies.

Functional analysis of both data sets did however show some similarities. There was a greater proportion of gene down-regulation in KC in both data sets. Furthermore, both studies found abnormalities in epithelium barrier with down-regulation of genes involved in tight junctions, gap junctions, adherens junctions and focal adhesion supporting a link to potential clinical changes. Both data sets revealed dysregulation of the Hedgehog, Wnt and Notch signaling pathways.

There were also significant differences in the results of both studies. For example, genes involved in the TGF beta signaling pathway tended to be up-regulated in our study but down-regulated in the Kabza *et al*., 2017 study. The TGF beta pathway is involved in wound healing and connective tissue remodeling and scarring^48^. It is plausible that this difference is due to the control groups in each study. As noted above, the controls from Kabza *et al*., 2017 included corneas from patients with corneal ulcers and scarring most likely with a greater degree of tissue damage and subsequent wound healing than KC patients (hence relative down-regulation of this pathway in KC). Differences may also be attributed to the fact that the tissue examined by Kabza *et al*., included both corneal epithelial cells and stromal cells; our study used only corneal epithelial cells^47^.

### Plasmolipin and Notch 1 signaling pathway in KC

Plasmolipin (PLLP) and Notch1 were validated by qPCR and Western blot which showed a significant reduction in both protein and gene expression in KC compared to controls. We also examined protein expression of two other proteins involved in Notch1 signaling pathways: Src/c-src and NICD^49,50^. Notch 1 signaling pathway activation requires multiple cleavage events which convert precursor Notch1 (p300, 300 kDa) to the active signaling protein^50^. The first cleavage occurs at the trans-Golgi network by Furin, where precursor Notch is cleaved to Notch1 receptor that is presented at the cell membrane^50^. Src is reported to regulate this cleavage event^50^. Our Western blot detected NTM region (120 kDa) of the Notch 1 receptor which showed significantly reduced expression in KC compare to control groups. Although we detected decreased expression of the *SRC* RNA in KC in our RNA-Seq study we see an increase in SRC protein in KC in the Western blot. This may indicate that the *SRC* mRNA is subject to miRNA regulation or that the protein life is extended. The increased protein expression of Src suggests that Notch1 receptor expression in KC may be linked to its reduced mRNA expression rather than Src regulation. After another two rounds of cleavage, NICD is generated and translocates to the nucleus to activate target genes^49,50^. No significant difference in expression of NICD was detected between KC and control epithelium at the protein or RNA level. One limitation could be that the antibody used in this study only recognized the NICD released by cleavage between Gly1753 and Val1754. Notch 1 signaling is a complex and critical pathway for many cellular activities, which is still being studied extensively. PLLP has recently been identified a regulating protein for notch 1 signaling^51^.

PLLP is a member of the MAL family containing 1 MARVEL domain. The MARVEL domain has been shown to be involved in cellular events such as vesicular transport and junction regulation^52^. PLLP function is not well-characterised, however it has been found to form cation-specific ion channels^53,54^, promote myelin synthesis, regulate polarised epithelial differentiation, tight junction formation and epithelial cell differentiation^51,55^. In addition, PLLP regulates zebrafish intestinal epithelial cell differentiation by regulating Notch signaling, and mutation of *pllp* reduces Notch1 expression^51^. These functions are also critical for normal corneal epithelium homeostasis and relevant to the GO terms associated with KC. PLLP may potentially play an important role in maintaining normal human corneal epithelial structure and function.

### Limitations

There are limitations to this study. At the time we commenced this study we did not expect to see a sex-related effect. This study revealed such a difference which in itself is a significant finding. However, it also reveals a weakness in that we have not been able to investigate KC in a mixed cohort. Accordingly, our study could be improved by having a mixed sex cohort of controls and KC patients. We expect that we would have seen more substantial agreement between our RNA-Seq and pPCR studies if we had been in a position to use a mixed cohort in both experiments. It should also be noted that our controls were myopic. Although this is not a corneal disease, it would be ideal to include an additional subgroup without refractive errors.

## Conclusion

Using epithelial samples taken from KC patients prior to undergoing collagen cross-linking, we investigated relatively early stage gene expression changes in KC. Our functional analysis of DEGs showed significant alterations of gene expression in cell-cell communication, cell junctions and cell signaling, which are consistent with morphological changes reported in KC epithelium. Genes associated with multiple metabolism mechanisms were also found to be associated with KC, suggesting the KC epithelium may undergo altered metabolism at an early stage. In particular, we found that both protein and gene expression of Notch1 and PLLP, were significantly down-regulated in KC compared to controls. Notch1 has not previously been linked to KC pathogenesis. PLLP was reported to be upregulated in KC corneal epithelium compared to normal through proteomic analysis^56^. Both previous finding and our paper suggested an abnormal expression of PLLP in KC, however our study showed PLLP was down-regulated in KC. The difference can be attributed to the sample types and preparation. Myopia rather than normal corneal samples were used as control in this study. We measured and compared PLLP expression in each sample, whereas the previous study used pooled sample^56^ and therefore may mask the individual differences. The role of PLLP in human corneal epithelium is unclear, however findings from studies in other tissues suggest that it could be important in maintaining normal corneal epithelial cellular activities and interact with the Notch 1 signalling pathway. Further studies are clearly warranted to confirm the functional role of these proteins in the pathogenesis of keratoconus.

## Methods

### Epithelial sample collection

All samples used in the study were obtained from the Vision Eye Institute, Chatswood, NSW, Australia with University of Sydney Human Research Ethics Committee approval (HREC 2013/1041). All procedures were in accordance with the Declaration of Helsinki. Informed consent was obtained from all participants prior to sample collection.

Control corneal epithelial tissues were collected from patients undergoing laser photorefractive keratectomy for mild myopia less than 6 diopters. Prior to the laser ablation, the cornea is prepared by the manual removal of the central 8 mm of epithelium. KC epithelial tissue was collected from patients undergoing collagen cross-linking to halt the progression of the disease. Similar to the refractive procedure, the central epithelial tissue was removed. In both procedures, the epithelium is routinely discarded. The removed epithelium was immediately submerged in RNALater (Qiagen, Germany) or RIPA buffer (Thermo Fisher Scientific, USA) in RNase-free 1,7 ml Eppendorf tubes, and subsequently used for RNA-Seq, quantitative PCR (qPCR), and Western blot.

A total of 53 samples (30 KC and 23 control) were used in this study (Table 1). Both contact lens wear and a clinical diagnosis of atopy were considered as preoperative conditions that may potentially influence our findings within the KC cohort. In our control cohort, 17 controls wore contact lenses and 6 wore glasses only. For KC patients, only 4 wore contact lenses, whereas the rest did not (Table S6). In addition, only 4 of 11 KC subjects confirmed with a diagnosis of atopy; 7 KC participants had an unconfirmed diagnosis. Control participants had no atopy except 1 case which remained unconfirmed (Table S6). Overall, contact lens wear and atopy were only reported in a small proportion of the participants sample, and the numbers were insufficient to conduct statistical analysis of the effects of these factors in our study of KC. As such, we did not include contact lens wear and atopy as covariates in this study.

### RNA isolation

RNeasy mini kit (Qiagen) was used to extract the RNA from tissues, following the manufacturer’s instruction with minor modifications. Briefly, RNALater was carefully removed from Eppendorf tubes containing tissues, and 300 μL of RLT containing 2-mercaptoethanol in a 100:1 ratio was added to each tube and the tubes were vortexed until the tissues were dissolved. Equal portions of 70% ethanol (300 μL) were added to each tube, vortexed, and centrifuged at 12,000xg for 20 s at room temperature (RT). The cartridge was washed wash buffer I (700 μL) at 12,000 g for 20 s followed by two more wash with wash buffer II (500 μL) at 12,000 g for 15 s. The cartridge was dried at 12,000xg for 1 min at RT and 30 μL of RNase-Free water was added to center of the cartridge and incubated at RT for 2 min. Purified RNA was eluted by centrifuging at 20,000xg for 2 min at RT and then stored at −80 °C for further use.

### RNA quality analysis

The sample quality was checked using a Nanodrop (Thermo Scientific, USA) and Bioanalyzer Nano RNA chip (Agilent, USA). Samples with RIN values above 8 were chosen for further analysis. A subset of the samples was also checked with the Xpose (Integrated Sciences, Australia) to assure that they did not contain any contaminants.

### RNA-Seq library preparation

Twenty samples were used for RNA-Seq including 10 KC and 10 controls. All 10 KC samples were from males, and there were 4 samples from females in the control group. The RNA samples were prepared using the TruSeq Stranded Total RNA Library Prep Kit (Illumina,USA) according to the manufacturer’s instructions. One μg of total RNA was used as input to the Ribozero rRNA-depletion step of the assay. The adapter-ligated cDNA was enriched using 12 cycles of PCR. The libraries were sequenced on a NextSeq. 500 (Illumina) using a 75 bp paired end read high output v2 run. This produced between 27 M and 60 M paired end reads per sample.

### RNA-Seq bioinformatics analysis

The samples were processed in 2 batches each with the same number of KC (n = 5 per batch) and control subjects (n = 5 per batch). We mapped the 75 nucleotide reads to the human genome (GRCh38) using TopHat2 (v 2.0.4)^56^, calling the Bowtie aligner (v 2-2.0.0-beta7)^57^, allowing up to 2 bp mismatches per read (default position). HTSeq-count (Python package HTSeq, python v 2.7.3) was used to generate counts of reads uniquely mapped to known and annotated genes using the Ensembl annotation file GRCh38.79.gtf (mode = union, –t = exon, –i = gene_name). The number of uniquely mapped reads varied between 41–50 million per sample in the first batch and 18–32 million per sample in the second batch. The count table of uniquely mapped reads was then used and differential expression was tested using the Bioconductor packages, edgeR (v3.16.0) and DESeq. 2 (v1.14.0).

In the edgeR analysis low count transcripts were excluded manually and only those genes with at least 1 count per million, in at least 5 samples, were used for analysis. This filtering retained 14585 of the original transcripts. A normalization factor was calculated using the trimmed mean of M values (TMM) method and the dispersion parameter for each gene was estimated as the Cox-Reid common dispersion method^58^. In the DESeq. 2 analysis, normalization was performed using the median-of-ratios method^59^. Dispersions were estimated using a Cox-Reid adjusted profile likelihood and the Wald test for significance was used. DESeq. 2 invokes automatic filtering to optimize the number of genes that have an adjusted p value below the threshold (default 0.1). Testing for differential expression employed a negative binomial generalized linear model for each gene. In both DESeq. 2 and edgeR batch and condition were used in the modelling. The Benjamini-Hochberg correction was used to correct for multiple comparisons with a false discovery rate (FDR) of 0.10 or 0.05 as indicated in this manuscript.

### cDNA synthesis and qPCR amplification

For qPCR validation, 9 KC samples including 7 independent samples and 2 from the RNA-Seq corhort was used (6 males and 1 female). A total of 10 control samples including 5 independent samples and 5 from the RNA-Seq cohort (3 males and 7 females) were used. Fifteen differentially expressed genes (DEGs) found by both edgeR and DESeq. 2 were amplified and normalized against 3 reference genes (Table 3). These genes represented a range of different functional categories including transcriptional regulation, cell communication, cell adhesion, cell migration, cell fate specification, transport, response to wounding, protein metabolism, post-translational modification, and apoptosis. cDNA was synthesized using iScriptTM gDNA Clear cDNA synthesis kit (Bio-Rad, USA) according to manufacturer’s instructions. A final 25 μL cDNA was generated from 1 μg of RNA per sample, and no reverse transcription control was generated for each corresponding cDNA. The diluted (5 fold) cDNA was used for qPCR reaction using SsoAdvanced^TM^ Universal SYBR^®^ Green Supermix (Bio-rad) and PrimePCR^TM^ SYBR^®^ Green Assay primers (Bio-Rad) (Table S7). For each qPCR reaction, 2 μL of diluted cDNA, 1 μL of PrimePCR^TM^ SYBR^®^ Green Assay primer, 10 μL of SYBR^®^ Green Supermix and 7 μL of nuclease-free water was used, and the reaction was performed in a CFX96^TM^ Real-Time System with C1000^TM^ Thermal Cycler (Bio-Rad) using the following cycling condition: 95 °C for 2 min, 40 cycles of 95 °C for 5 sec, and 60 °C for 30 sec, followed by 95 °C for 5 sec. A melting curve analysis was included as the last step with temperature ramping from 65 °C to 95 °C increasing by 0.5 °C every 5 sec. NRT controls were tested with each primer to check for gDNA contamination, and all assays were performed in duplicates. The results were analyzed by CFX Manager 3.1 (Bio-Rad) using ΔΔCT method with three reference genes *GUSB*, *TBP* and *HPRT1*.

### Western blotting analysis

Independent samples were used for Western blot including 13 KC (8 males and 5 females) and 8 control (2 male and 6 females) samples. Proteins were extracted by firstly homogenizing the collected epithelial tissues in RIPA buffer (Sigma, USA) supplemented with cOmplete™ Protease Inhibitor Cocktail Tablets (Roche, USA) for 10 min using a homogeniser, followed by centrifugation at 12,000 × g for 15 min and 4 °C. The supernatant was removed, aliquoted and stored at −80 °C until use. Protein concentration was measured using a FluroProfile^®^ kit (Sigma-Aldrich, USA) as previously published^8^. Sixty micrograms of total protein from each sample was used.

Western blotting was performed as previously published^60^. Samples were tested for the expression of Notch1, cleaved Notch1 (NICD), plasmolipin (PLLP) and proto-oncogene tyrosine-protein kinase (Src), and GAPDH and Pan-actin were used as the loading control (Table S8). HRP conjugated anti-rabbit IgG secondary antibody (Millipore) was used at 1 in 25,000 dilution. The band volume for each protein (with background subtracted) were analysed using the GeneTool software (Syngene, UK), and the ratio of target proteins/GAPDH was calculated. A two-tailed Student’s t-test was used to determine the significance between KC and control samples (p < 0.05 is considered as significant).

### Comparison of corneal epithelium RNA-Seq (current study) with corneal button RNA-Seq

Recently, Kabza *et al*., (2017) published a RNA-Seq study of KC in which they compared gene expression in the corneal button (corneal epithelium, stroma and endothelium) taken from KC patients to expression in the corneal button of patients unaffected by KC, but who were undergoing corneal transplantation (designated the control group). Notably, these controls included patients being treated for conditions such as bullous keratopathy, corneal scarring, corneal ulcers and perforations^47^. We took the count tables available at GSE77938 (GSE77938 discovery gene counts.txt and GSE77938 replication gene counts.txt) and used edgeR to carry out differential expression on this data set, using the same parameters in our study, as described above and including the batch effect in our Generalised Linear Model design.

### Functional analysis

We analyzed the differentially expressed genes for functional annotations using the goana function from the limma Bioconductor package. We applied this function to the MArrayLM fit object derived from the preceding edgeR analysis. The Entrez Gene IDs associated with any gene ontology term was used as the background and an FDR of 0.05 was imposed. We used goana for gene ontology enrichment analysis of our data set and also the Kabza *et al*. data set.

The fry function from the limma package was used to perform gene set tests on the DGElist object generated in the edgeR analysis. We interrogated the human-c2_v5 data set downloaded from the WEHI site (http://bioinf.wehi.edu.au/software/MSigDB/index.html). Our RNA-Seq data was weighted by the average logCPM values of each gene. We extracted KEGG gene sets from this analysis using an FDR cut-off of 0.01. We used this gene set analysis methodology to analyze our data set and also the Kabza *et al*. data set.

### Data Availability

The datasets generated during and/or analysed during the current study are available from the corresponding author on reasonable request.

## Electronic supplementary material

Supplementary Information

Table S1

Table S2

Table S3

Table S4

Table S5
