# Author Correction: RNA-Seq analysis and comparison of corneal epithelium in keratoconus and myopia patients

**DOI:** 10.1038/s41598-021-98363-4

**Published:** 2021-09-30

**Authors:** Jingjing You, Susan M. Corley, Li Wen, Chris Hodge, Roland Höllhumer, Michele C. Madigan, Marc R. Wilkins, Gerard Sutton

**Affiliations:** 1 Save Sight Institute, Sydney Medical School, University of Sydney, Sydney, Australia; 2 School of Optometry and Vision Science, University of New South Wales, New South Wales, Australia; 3 School of Biotechnology and Biomolecular Science, NSW System Biology Initiative, University of New South Wales, New South Wales, Australia; 4 Lions NSW Eye Bank, Sydney, Australia; 5 Vision Eye Institute, Chatswood, New South Wales Australia; 6 University of the Witwatersrand, Johannesburg, South Africa; 7 The Cornea Foundation, Johannesburg, South Africa

Correction to: *Scientific Reports* https://doi.org/10.1038/s41598-017-18480-x, published online 10 January 2018

In the original version of this Article, a relevant paper on the detection of PLLP in human corneal epithelium during KC pathogenesis was not cited. This article is now cited as Ref 56 and discussed. As a result, in the Conclusion,

"Notch1 and PLLP have not previously been linked to KC pathogenesis. PLLP has not been previously reported to be expressed in human corneal epithelium, although its roles in other tissues suggests it could play a critical role in normal corneal epithelial cellular activities, and interact with the Notch1 signaling pathway."

now reads:

"Notch1 has not previously been linked to KC pathogenesis. PLLP was reported to be upregulated in KC corneal epithelium compared to normal through proteomic analysis^56^. Both previous finding and our paper suggested an abnormal expression of PLLP in KC, however our study showed PLLP was down-regulated in KC. The difference can be attributed to the sample types and preparation. Myopia rather than normal corneal samples were used as control in this study. We measured and compared PLLP expression in each sample, whereas the previous study used pooled sample^56^ and therefore may mask the individual differences. The role of PLLP in human corneal epithelium is unclear, however findings from studies in other tissues suggest that it could be important in maintaining normal corneal epithelial cellular activities and interact with the Notch 1 signalling pathway."

Subsequent references in the Article have been renumbered accordingly. The original Article has been corrected.

## References

[56] Chaerkady R, Shao H, Scott SG, Pandey A, Jun AS, Chakravarti S (2013). The keratoconus corneal proteome: loss of epithelial integrity and stromal degeneration. J Proteomics.

