# Supplementary material for: RNA-Seq analysis and comparison of corneal epithelium in keratoconus and myopia patients
Source: Sci Rep. 2018 Jan 10;8:389. doi: 10.1038/s41598-017-18480-x (PMC5762683; doi:10.1038/s41598-017-18480-x)
Supplement: Supplementary file 1 — Supplementary Information [file 41598_2017_18480_MOESM1_ESM.pdf]

RNA-Seq analysis and comparison of corneal epithelium in keratoconus and myopia patients

Jingjing You[1,2*], Susan Corley[3], Li Wen[1], Chris Hodge[4,5], Roland Höllhumer[6,7], Michele C. Madigan[1,2], Marc R. Wilkins[3], Gerard Sutton[1,4,5]

1. Save Sight Institute, Sydney Medical School, University of Sydney, Australia
2. School of Optometry and Vision Science, University of New South Wales, Australia
3. School of Biotechnology and Biomolecular Science, NSW System Biology Initiative, University of New South Wales, Australia
4. Lions NSW Eye Bank, Sydney, Australia
5. Vision Eye Institute, Chatswood, New South Wales, Australia
6. University of the Witwatersrand, Johannesburg, South Africa
7. The Cornea Foundation, South Africa

Jingjing You and Susan Corley contribute equally to this work and are joint first authors

*Corresponding author:

Jingjing You

jing.you@sydney.edu.au

Table S6: Contact lens wearing and diagnosis of atopy for corneal epithelial samples used in this study.

Sample ID	Condition	Gender	Age	Contact lens wear	Atopy
1	KC	M	17	No	Yes
2	KC	M	39	No	No
3	KC	M	40	No	No
4	KC	M	22	No	Yes
5	KC	M	28	No	No
6	KC	M	16	No	No
7	KC	M	25	No	No
8	KC	M	22	No	No
9	KC	M	26	Yes	No
10	KC	M	35	No	No
11	Control	F	35	Yes	No
12	Control	M	32	Yes	No
13	Control	F	39	Yes	No
14	Control	F	35	Yes	No
15	Control	M	30	No	No
16	Control	M	33	Yes	No
17	Control	M	27	Yes	No
18	Control	M	43	Yes	Unconfirmed
19	Control	F	23	Yes	No
20	Control	M	23	No	No
21	Control	F	44	No	No
22	Control	F	34	Yes	No
23	Control	F	46	Yes	No
24	Control	F	36	No	No
25	Control	F	35	Yes	No
26	KC	F	43	No	No
27	KC	M	13	No	No
28	KC	M	21	No	Yes
29	KC	M	17	No	No
30	KC	M	32	No	No
31	KC	M	18	No	Yes
32	KC	M	20	No	No
33	KC	F	26	Yes	No
34	KC	M	24	Yes	Unconfirmed
35	KC	M	30	Yes	Unconfirmed
36	KC	F	38	No	Unconfirmed
37	KC	M	23	No	No
38	KC	F	36	No	No
39	KC	M	24	No	No
40	KC	M	19	No	No

41	KC	F	24	No	Unconfirmed
42	KC	M	22	No	Unconfirmed
43	KC	M	28	No	Unconfirmed
44	KC	F	25	No	Unconfirmed
45	KC	M	18	No	No
46	control	F	31	No	No
47	control	F	37	Yes	No
48	control	F	39	Yes	No
49	control	M	47	Yes	No
50	control	F	45	No	No
51	control	F	29	Yes	No
52	control	F	29	Yes	No
53	control	M	32	Yes	No

Table S7: PrimePCR^{TM} SYBR$^{®}$ Green Assay primers used in this study.

**Gene Symbol**	**Ensembl Gene ID**	**Amplicon Context Sequence**	**Unique Assay ID (Bio-rad)**	**Efficiency (%)**
NOTCH1	ENSG00000148400	GCTGCCGCAGGTACGAGCGTCATTCTCACACGTGGCGCCCTCGAAGCCCGCAGGGCACTTGCAGATGAACCCGCGGGCGGTGTTGGAGGCCACGGCGCAGGTGCCCCCATTCTTGCAGGGCTTGCCTTTGCAGCCATTGATGACGGACTCGCAGCGGCGCCCGGTGTGACCA	qHsaCID0011825	96
PLLP	ENSG00000102934	CAGGCCTGGTAGCTGAAGAAGGCACTCACTCCATAGGCGATCATCACCAAACACGCAAAGAACGAGGCAGCCGCGCGCTGGTTATAAGGCCGGGTGCCCCTCAGGGATGTCAGGTCAACTGCCGCAGAGCAGGCGATGAAGGCGGTGATGTAGAGAACGGTGGCGCTGATGTTAAAGAT	qHsaCID0008089	99
LY6D	ENSG00000167656	CAAAACAGAAGGAGTGTGAAATCCGGGGATCCACAGGGCTTCTGTCCTCCACCTTCCATGCAGCTGGGGGCTGCATCCTCTGTGGGGTGGCTTCATCCTCTGTGGGGTCTGTGGGGCCTGCTCCAAGTCATCAGCATTCCATGCCCACCTGGACCTGGTCC CAGACTTTCGG	qHsaCED0007859	103
CAMKK1	ENSG00000004660	ACTTACACCAGTAGGTTTCCTGGAGCAGACATGGATCGCTCTTCCCTCCGTGCTTGGGGCTCAAACGGGTTCCCAAAGGAACGCTTCCTCAGCATGGACTTCACCAGGATCACCGTGGTCCAGCTGGGGATGAGCCTGACTGAGTTCTTAACC	qHsaCID0016669	99
CISH	ENSG00000114737	CCCCGGGGCCTGCGCCACAGTGAGACCAGCTCTATGCAGATACAGCTCTGTCCTGTGAGGGGGTGAGACACAGGCTCTGCTGGGGACTGAGGCTCCGGCTGCATTTTCTTACCTATTGTAGTTGTCCATCCTCCCC	qHsaCED0043561	98

PLEKHG3	ENSG00000126822	GCCTCCGGATCAAGAGCAACAAGCCAGTGATGGCCAGGCCACCACTGCAGTGGGAAAAGGTGGCCCCTGAGAGGGATGGGAAGAGCCCCACTGTGCCCTGTCTACAGGAAGAGGCTGGAGAGCCATTAGGTGGCAAAGGTAAGAGGAAGCCGGTGCTGTCTCTATTTGACTATGAGCAGCTGATGGCCCAGGAGCACAGCCCTCCCAAGCCCT	qHsaCID0014586	98
NR1D1	ENSG00000126368	AGGAGCCACTGGAGCCAATGTAGGTGATGACGCCACCTGTGTTGTTGTTGGAGTCCAGGGTCGTCATGTCTTCACCAGCTGAGAGCGGTCATTCAAACTGGACCTTGACTCAAACTAGAGGTTGCGA	qHsaCID0015596	100
NINJ1	ENST00000375446	GACCCCGAAGGCCGTGATGAAGATGTTGACTACCACGATGATGAACACCAGGCCCGTGGCCAGGTTGTTGAGGAAGTCCAGCTTGGCGTGCTTG	qHsaCED0004249	101
Scara5	ENSG00000168079	TCGAAGTCCAGGCAATCCAGGGATGCCAGGCCTGCCTTCCTTTCCTTCATCCCCCTGATCACCTTTGGGTCCCGGTGGCCCTTTCGCGAGGGAGATGTTCCGCAGTG CGATG	qHsaCID0022568	101
EPHB4	ENSG00000196411	CGTGTAGGTGGGATCGGAAGAGTTCTCCTCCAGGAATCGGGAAAGGCCAAAGTCAGACACTTTGCAGACGAGGTTGCTGTTGACTAGGATGTTGCGAGCAGCCAGGT CTC	qHsaCED0003993	98
MAGI3	ENSG00000081026	CTCCGAGGGGGGAAGGAGTACAACATGGGGCTGTTCATCCTTCGTCTTGCTGAAGATGGTCCTGCCATCAAAGATGGCAGAATTCATGTTGGTGACCAGATTGTTGAAATCAATGGGGAACCTACACAAGGAATCACACATACTCGAGCAA	qHsaCID0006685	98
PHLDB1	ENSG00000019144	TCTATTTCCAGGCCATTGAGGAAGTGTACTACGACCACCTGCGCAGTGCAGCCAAGAAGAGGTTTTTCCGCTTCACTATGGTGACTGAGAGCCCGAACCCAGCCCTCACCTTCTGC	qHsaCID0010418	103

		GTAAAGACCCATGACCGGC TGTACTACATGGTGGCCCC ATCTGCAG AGGC		
PALM	ENSG0000 0099864	CCGCGAGCCTTCGCTGAAG TGCCCTTGCTATAACCCCCT CTGCTTCTGGTGTGTGACG AGGCCCCCGATGTTCTTGA TTTTCCCAGAGAAGCAAATA AACAGCGTGAACAGCCCCA AAAATTAGCC	qHsaCED00 36361	96
FMO3	ENSG0000 0007933	GGTTGGACAGGACGTAGAC ACACAGAAGAAAAGAAGAC AAAGAACGGGTAGGAAAAT TAAAAAGGTTACCATGGGG AAGAAAGTGGCCATCATTG GAGCTGGTGTGAGTGGCTT GGCCTCCATCA	qHsaCID00 08817	100
ZNF100	ENSG0000 0197020	AATTTTGTAAAAATTATTCTT ATAATCACAGACCAGCTCAC ATAATGAATACTTCATAATCT GTAAAATATTTATATGAAAAA AGATACAAAATAATTAGGGG TCTATCATGAGTACCAGGCA AGTATAAACAGAATTTTCAT GGGGAGATTCAGAACTATA AGCCAC	qHsaCED00 34358	100
Reference genes				
TBP	ENSG0000 0112592	AAATATTGTATCCACAGTGA ATCTTGGTTGTAAACTTGAC CTAAAGACCATTGCACTTCG TGCCCGAAACGCCGAATAT AATCCCAAGCGGTTTGCTG CGGTAATCATGAGGATAAG AGAGCCACGAACCACGGCA CTGATTTTCAGTTC	qHsaCID00 07122	101
HPRT1	ENSG0000 0165704	GACACTGGCAAAACAATGC AGACTTTGCTTTCCTTGGTC AGGCAGTATAATCCAAAGAT GGTCAAGGTCGCAAGCTTG CTGGTGAAAAGGACCCCAC GAAGTGTTGGATATAAGCCA GAC	qHsaCID00 16375	105
GUSB	ENSG0000 0169919	ACCAAGAGTAGTAGCTGTTC AAACAGATCACATCCACATA CGGAGCCCCCTTGTCTGCT GCATAGTTAGAGTTGCTCAC AAAGGTCACAGGCCGGGAG GGGTCCAAGGA	qHsaCID00 11706	103

Table S8: Antibodies used for Western blot

Protein	Host Species	Catalogue/Company	Dilutin*
Notch 1	Rabbit	4380s/Cell Signaling Technology, MA, USA	1:500
Cleaved Notch 1	Rabbit	4147T/Cell Signaling Technology	1:500
Src	Goat	2108/Cell Signaling Technology	1:500
PLLP	Rabbit	MBS7003196/Resolvingimages P/L VIC, Australia	1:500
GAPDH	Rabbit	9585/Abcam, Cambridge, UK	1:2500
Pan-actin	Mouse	MA5-11869/Neomarker, CA USA	1:400

* As not all concentration of antibodies were provided by company, dilution is listed here.

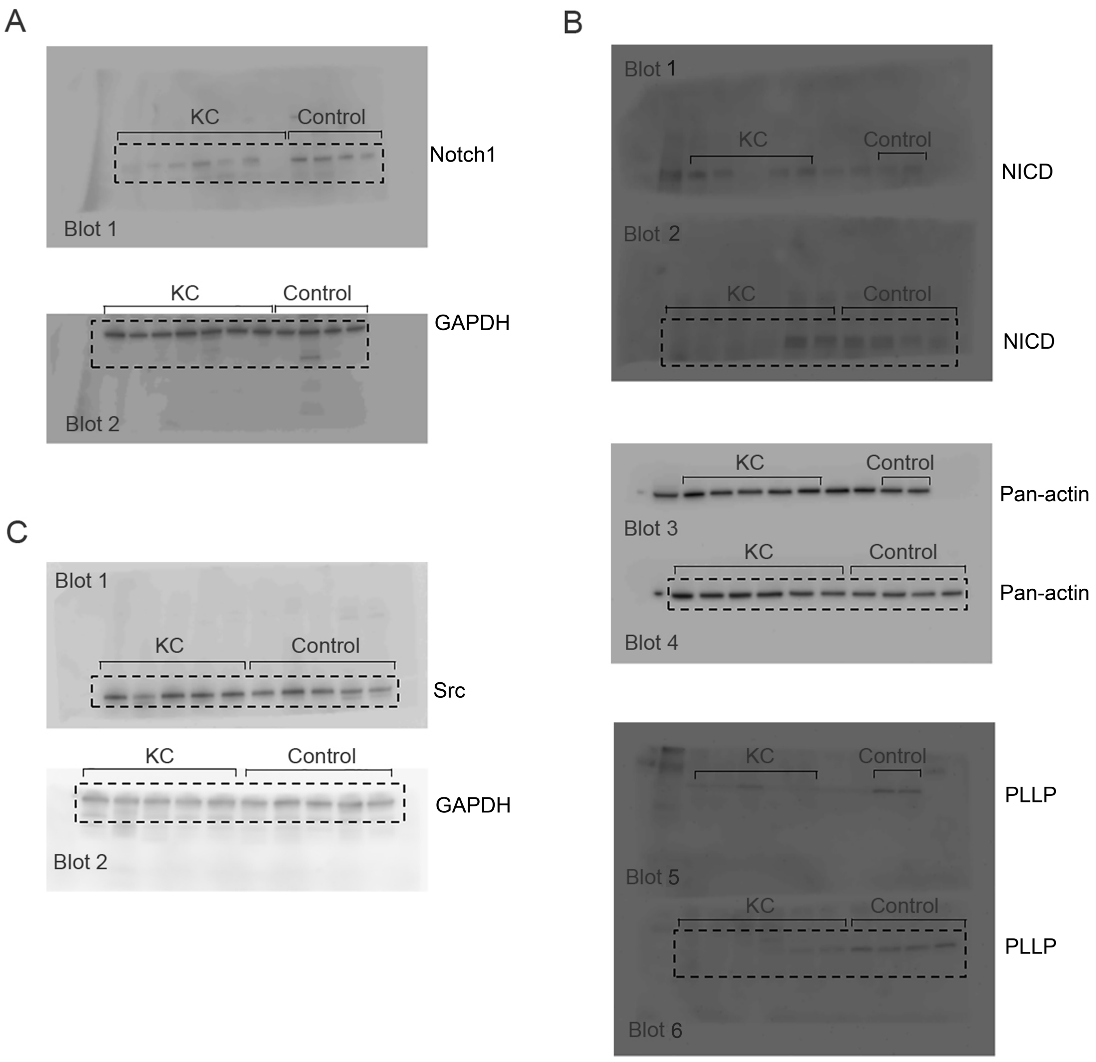

Supplementary Figure S1: Full-length blots for each protein quantified. Dashed line-enclosed regions represent the cropped region in Figure 4. A: Blot 1 and 2 were from the same blot. Blot 1 was probed with Notch 1 antibody and Blot 2 was probed with GAPDH antibody. B: Blots 1, 3 and 5 were from the same blot; Blots 2, 4 and 6 were from the same blot. NICD was probed on Blot 1 and 2, Pan-actin was probed on Blots 3 and 4, and PLLP on Blots 5 and 6. C: Blot 1 and 2 were from the same blot, Src was detected on Blot 1 and GAPDH on Blot 2.
